# A Retrospective Assessment of Changes in Stroke Risk-Related Biomarkers in Individuals with Prediabetes from Durban, South Africa: Preliminary Findings

**DOI:** 10.3390/cimb47110884

**Published:** 2025-10-24

**Authors:** Yerushka Naicker, Andile Khathi

**Affiliations:** Department of Human Physiology, School of Laboratory Medicine and Medical Sciences, College of Health Sciences, University of KwaZulu-Natal, Durban 3629, South Africa

**Keywords:** prediabetes, type 2 diabetes mellitus, stroke, Durban, South Africa

## Abstract

Type 2 diabetes mellitus (T2DM) is a chronic metabolic disorder that significantly increases the risk of stroke, with prediabetes serving as an intermediate stage marked by similar pathophysiological mechanisms such as inflammation and vascular dysfunction. This study investigated the relationship between prediabetes and stroke-related biomarkers in individuals aged 25–45 years in Durban, South Africa. After obtaining ethical approval, a retrospective analysis was performed on blood samples from 100 participants recruited from King Edward Hospital and Inkosi Albert Luthuli Central Hospital. Participants were classified as non-prediabetic (*n* = 30), prediabetic (*n* = 35), or type 2 diabetic (*n* = 35) according to ADA criteria. Plasma concentrations of C-reactive protein (CRP), interleukin-6 (IL-6), fibrinogen, D-dimer, calcium binding protein (S100B), glial fibrillary acidic protein (GFAP), and neuron-specific enolase (NSE) were measured using enzyme-linked immunosorbent assay (ELISA). It is important to note that none of the participants had confirmed stroke events; these biomarkers were assessed as surrogate indicators of stroke risk. Statistical analyses included one-way ANOVA with Tukey–Kramer tests and Pearson’s correlations. Biomarker concentrations were significantly elevated in prediabetic individuals compared to non-prediabetic controls, with levels further increasing in T2DM. Strong positive correlations were observed between S100B and both HbA1c (r = 0.75, *p* < 0.0001) and fasting glucose (r = 0.75, *p* < 0.0001). These findings suggest that inflammatory, coagulation, and neurovascular biomarkers, particularly S100B, may indicate early stroke risk in prediabetes. Further investigation into these biomarkers could improve early detection strategies and stroke prevention efforts in at-risk populations.

## 1. Introduction

Type 2 diabetes mellitus (T2DM) is a chronic metabolic condition characterised by insulin resistance that results in elevated blood glucose levels [1]. The prevalence of T2DM is rapidly increasing in South Africa, consequently placing a significant burden on the South African health system [2]. A systematic review published in 2021 estimated that the pooled prevalence of T2DM in South Africans aged 25 years and older was 15.25% [3]. In South Africa, T2DM ranks second among the top 10 leading natural causes of death, thus emphasising the magnitude of the diabetes epidemic [4]. Heart failure and stroke are complications frequently associated with T2DM and may become life-threatening [5,6,7]. Stroke is not an independent disease but can be triggered by a multitude of risk factors and disease mechanisms [8]. A national burden of disease study in 2000 reported that T2DM accounted for approximately 10% of all stroke cases [2,9]. According to studies, people with T2DM have a higher risk of stroke than those without T2DM [7,10]. Notably, individuals with diabetes mellitus who experience symptomatic cerebral ischaemia have been shown to have a higher risk of recurrent stroke, and the female sex is a demographic factor associated with a worse prognosis [11]. These findings highlight the complexity of stroke pathophysiology and suggest that biological factors, including sex-related differences and pre-existing metabolic disturbances, may influence disease progression and prognosis.

The pathophysiology of stroke involves the abrupt interruption of blood supply to the brain, resulting in the brain being deprived of oxygen and causing damage to brain cells [12]. A stroke can manifest as either ischaemic, which is due to a lack of blood flow, or haemorrhagic, which is due to bleeding in a specific area. These disruptions can lead to a range of neurological impairments, such as paralysis, speech difficulties, and cognitive deficits, depending on the location and extent of the brain damage [8]. In T2DM, hyperglycaemia leads to oxidative stress, inflammation, and endothelial dysfunction, increasing vascular damage and stroke risk [13]. Both T2DM and stroke share common inflammatory pathways [14]. Elevated inflammatory markers contribute to the onset of a stroke, while the stroke event itself also triggers a subsequent inflammatory response, resulting in increased levels of circulating inflammatory mediators [15]. Elevated C-reactive protein (CRP) and interleukin 6 (IL-6) levels can signal inflammation, which plays a role in stroke development and progression [16,17,18]. Increased fibrinogen and D-dimer levels can indicate inflammation and blood clotting, both of which are associated with stroke risk and damage [19,20,21]. Neuron-specific enolase (NSE), glial fibrillary acidic protein (GFAP), and calcium-binding protein S100B are biomarkers of brain injury. The concentrations of these biomarkers are increased after stroke and help to determine the severity of brain injury, with NSE being a marker of neuronal death, GFAP for astrocytic injury and S100B for brain cell injury. Together these biomarkers have been shown to help distinguish between stroke types, to assess the severity of the stroke and predict the outcomes including the potential for functional recovery in individuals with diabetes [22,23,24].

Given that metabolic disturbances such as hyperglycaemia and insulin resistance significantly influence stroke risk and outcomes in patients with diabetes, it is important to consider whether similar processes may already be active before the onset of overt disease.

Before the onset of T2DM, moderate insulin resistance causes a gradual increase in glucose levels referred to as prediabetes [25]. Prediabetes or intermediate hyperglycaemia is a severe health condition characterised by blood glucose concentration higher than normal but below the established threshold for T2DM [26]. The global prevalence of prediabetes is estimated at 318 million [27]. A systematic review and meta-analysis conducted in 2022 found that the prevalence of prediabetes among South Africans aged 25 to 45 was 15.56% [25]. Another retrospective study conducted in Durban revealed a concerningly high prevalence of prediabetes among individuals aged 25–45 years [28]. This is alarming as research has shown that individuals with prediabetes have an increased risk of stroke and progression to T2DM [29]. Prolonged elevated blood glucose in the prediabetic state can upregulate markers of chronic inflammation and contribute to the generation of reactive oxygen species (ROS) [30]. Excessive ROS generated can damage the brain tissue and is an important pathological mechanism of stroke [31]. Despite this growing prevalence, there is a notable absence of research investigating stroke-related biomarkers specifically in South African prediabetic populations. Most existing studies were conducted in Western or Asian contexts, which may not be fully generalisable given the distinct genetic, socioeconomic, and healthcare characteristics of African populations. Although a scoping review by Van Vuuren (2022) identified several blood biomarkers with potential prognostic value for stroke outcomes, and other studies have explored the prevalence of prediabetes in stroke patients, none have specifically assessed stroke-related biomarkers in individuals with prediabetes within the South African context [29,32,33]. Measuring these biomarkers in the prediabetic stage is essential because this phase presents a window of opportunity for early intervention before irreversible vascular and neurological damage occurs. Identifying alterations in inflammation, coagulation, and neurovascular injury markers could enable the development of predictive tools for stroke risk stratification. This highlights the urgent need for further investigation to facilitate early detection strategies and preventive measures for this demographic. Previous studies assume prediabetes is an intermediate metabolic state primarily based on associations with later diabetes or cardiovascular outcomes. In contrast, this study directly investigated early neurovascular, inflammatory and coagulation changes in young South African adults, a population in which prediabetes is increasingly prevalent but remains underrepresented in biomarker research. To our knowledge, this is among the first studies to comprehensively characterise these biological changes in a prediabetic cohort, offering novel insights into stroke risk at an earlier disease stage. Therefore, the aim of this study was to evaluate stroke risk in individuals with prediabetes (aged 25–45 years) in Durban, South Africa, using plasma biomarkers of inflammation, coagulation and neurovascular injury.

## 2. Materials and Methods

### 2.1. Ethical Approval

Approval was obtained from the UKZN Biomedical Research Ethics Committee (BREC) BE266/2019. Informed consent for storage of samples and potential studies was obtained from each participant.

### 2.2. Sample Collection

These samples were retrospectively obtained as leftover clinical specimens from a previous ethically approved study and stored in a biobank for future research. A convenience sampling method was used.

These samples, originally collected as part of the previous study, were collected from individuals between the ages of 25–45 years, from King Edward Hospital and Inkosi Albert Luthuli Central Hospital in Durban, South Africa.

The selection of samples was performed according to predefined inclusion and exclusion criteria. The blood samples were collected from adult individuals of all ethnicities and both males and females, aged between 25 and 45 years. Included were samples obtained from individuals clinically diagnosed as non-prediabetic, prediabetic, or with type 2 diabetes mellitus, based on established diagnostic criteria. All participants were systematically screened, and individuals with a history of traumatic brain injury, hypertension, blood disorders, depression, HIV, and kidney/heart/liver diseases were excluded, regardless of their glycaemic status.

The inclusion criteria focused on individuals aged 25–45 years to target a younger adult population at early stages of prediabetes and T2DM, where early biomarker changes might be detectable. We know that inflammation and coagulation are common pathways involved in many illnesses; therefore, to isolate the specific relationship between prediabetes and stroke risk, individuals with conditions known to affect these pathways, such as HIV infection, hypertension, traumatic brain injury, and chronic organ diseases, were excluded. This exclusion helped minimise confounding factors and strengthen the validity of associations observed between glycaemic status and stroke-related biomarker alterations.

Relevant clinical data, including age, sex, fasting glucose, and HbA1c levels, were measured at the time of sample collection and recorded for the purpose of this study. Lifestyle factors such as smoking, alcohol consumption, diet, and BMI were not recorded in the retrospective dataset.

### 2.3. Sample Size Calculation

The following formula was used to determine the appropriate sample size for this study.

$$n=\frac{z^{2}\times P\times \left(1-P\right)}{E^{2}}$$

where

• *n* is the required sample size.
• Z is the Z-score corresponding to the chosen confidence level.
• P represents the assumed prevalence.
• E is the desired margin of error.

$n=\frac{1.96^{2}\times 0.10\times (1-0.10)}{0.05^{2}}$

*n* = 138.2976

*n* = 138

For this study, a 95% confidence level was used, with a corresponding Z-score of 1.96. The assumed prevalence (P) of stroke cases due to diabetes in South Africa is 10% and a margin of error (E) of 5% was considered [2]. Using these parameters, the required sample size was calculated to be 138.

The G*Power software version 3.1.9.7 was also used to calculate the required sample size needed to achieve a power of 0.95, which was determined to be 45 [34]. The input parameters and the corresponding output are shown below.

Test Family: F tests

Statistical test: ANOVA: Repeated measures, within factors

Type of power Analysis: A priori: Compute required sample size

Input:

Effect size f = 0.25

α err prob = 0.05

Power (1-β err prob) = 0.95

Number of groups = 3

Number of measurements = 3

Corr among rep measures = 0.5

Nonsphericity correction ε = 1

Output:

Noncentrality parameter λ = 16.8750000

Critical F = 3.1051566

Numerator df = 2.0000000

Denominator df = 84.0000000

Total sample size = 45

Actual power = 0.9597015

Although the formula-based calculation suggested a sample size of 138 and the GPower analysis indicated that 45 samples were sufficient for 95% power, a total of 100 samples were available for analysis due to the limited availability of stored samples that met the inclusion criteria. This limitation was primarily due to feasibility constraints related to sample accessibility and quality. This study collected a total of 100 blood samples from various patients. Based on HbA1c parameters, the samples were categorised into three groups: non-prediabetic (NPD, *n* = 30), prediabetic (PD, *n* = 35), and type 2 diabetes mellitus (T2DM, *n* = 35). Due to limited plasma volumes and assay cost constraints, subsets of samples were randomly selected for specific biomarker analyses: CRP, fibrinogen, and S100B (*n* = 20 per group); IL-6, D-dimer, GFAP, and NSE (NPD *n* = 10, PD/T2D *n* = 15). Random selection ensured representative sampling and minimised selection bias.

### 2.4. Study Design

This study employed a retrospective cross-sectional design using stored blood samples collected from hospital patients. Upon arrival at the university, samples were categorised into groups as per criteria outlined by the American Diabetes Association. Blood glucose levels and glycated haemoglobin levels, recorded at the time of admission to the hospital or at the time of recruitment were used to divide the samples into a non-prediabetic group, a prediabetic group, and a type 2 diabetic group. The ADA criteria provide a clear guideline designed to diagnose prediabetes, as depicted in the table below (see Table 1).

Thereafter, the blood samples were subjected to centrifugation (Eppendorf centrifuge 5403, Hamburg, Germany) at 4 °C, 503× *g* for a duration of 15 min to isolate plasma. Following centrifugation, these plasma samples were preserved at −80 °C inside a Bio Ultra freezer (Snijers Scientific, Tilburg, The Netherlands) until biochemical analysis was conducted.

### 2.5. Biochemical Analysis

CRP, IL-6, Fibrinogen, D-dimer, S100B, NSE and GFAP concentrations were determined using their respective ELISA kits (Elabscience Biotechnology Co., Ltd., Houston, TX, USA) according to manufacturer’s specifications. Briefly, the ELISA kits contained micro-ELISA plates that were coated with antibodies. One hundred microliters of samples were placed into designated wells, followed by a 90 min incubation at 37 °C. The liquid was then discarded and one hundred microliters of biotinylated detection antibody working solution was promptly added to each well, with an additional incubation of 60 min at 37 °C, after which the unbound constituents were removed using the wash buffer supplied. Post-washing, 100 µL of horseradish peroxidase (HRP) was introduced into the plates and left to incubate for 30 min at 37 °C. Another wash step was performed to eliminate unbound substances. Next, 90 µL of substrate reagent was added to the wells, followed by an additional 15 min of incubation at 37 °C. Fifty microliters of a stop solution was added to the wells to stop the reaction and allow for measurement. Optical densities were then measured at a 450 nm wavelength using a nano spectrophotometer (BMG Labtech, Passau, Germany). The CRP, IL-6, Fibrinogen, D-dimer, S100B, NSE and GFAP concentrations in the samples were extrapolated from their respective standard curves.

To ensure objectivity during biochemical analysis, all plasma samples were coded and laboratory personnel conducting the ELISA were blinded to participant group assignments. While samples were grouped for batch testing, the laboratory technicians were not informed whether the batch belonged to the NPD, PD or T2D category. Relevant clinical data (including age, sex, name, HbA1c and glucose levels) were stored separately in Excel spreadsheets. Each sample was allocated a unique code, which was labelled directly on the sample tube. Additionally, all assays were performed in duplicates to enhance the accuracy and reliability of the results.

### 2.6. Statistical Analysis

All the data obtained from the experiment were expressed as means ± SEM. Statistical comparisons were performed using Graph Pad InStat Software (version 5.00, GraphPad Software, Inc., San Diego, CA, USA). The differences between the 3 groups were determined using analysis of variance (ANOVA) followed by the Tukey–Kramer multiple comparison test. A value of *p* < 0.05 was considered statistically significant. Correlation analyses were conducted using Pearson’s correlation coefficient to examine the strength and direction of the relationship between variables. A *p*-value of <0.05 was considered statistically significant.

Sensitivity analyses were not required in this study because key potential confounders, particularly HIV infection, were addressed at the design stage. Individuals with known comorbidities, including HIV infection, were excluded from the sample to reduce bias linked to inflammation and coagulation abnormalities. The study specifically targeted participants aged 25–45 years to control for age-related variability. While factors such as smoking, alcohol use, and BMI were not assessed in the present analysis, they are acknowledged as important variables and are recommended for inclusion in future research to further refine understanding of the association between biomarker levels and stroke risk.

## 3. Results

### 3.1. Demographic and Clinical Characteristics of Study Participants

Table 2 summarises the demographic and clinical characteristics of participants according to glycaemic status. Age data were normally distributed (Shapiro–Wilk test) and are presented as mean (95% CI), whereas Impaired Fasting Glucose data were not normally distributed and are presented as median (IQR). An upward trend in age was noted, with a mean of 36.1 years in the non-prediabetic group and 40.3 years in the type 2 diabetic group. This suggests a possible association between increasing age and glycaemic deterioration. Sex distribution varied across groups, with females comprising 60% of the non-prediabetic group and 68.6% of the type 2 diabetic group, while males were more common in the prediabetic group at 57.1%. Statistical comparisons revealed that median fasting glucose levels were significantly higher in the prediabetic group compared to the non-prediabetic group (*p* < 0.001) and further significantly elevated in the type 2 diabetic group compared to both the non-prediabetic and prediabetic groups (*p* < 0.001). Similarly, mean HbA1c levels showed a stepwise increase, with significant differences between all groups (*p* < 0.001). These results indicate a clear progression of glycaemic impairment across the study groups.

### 3.2. C-Reactive Protein (CRP) Concentration

**Figure 1:**
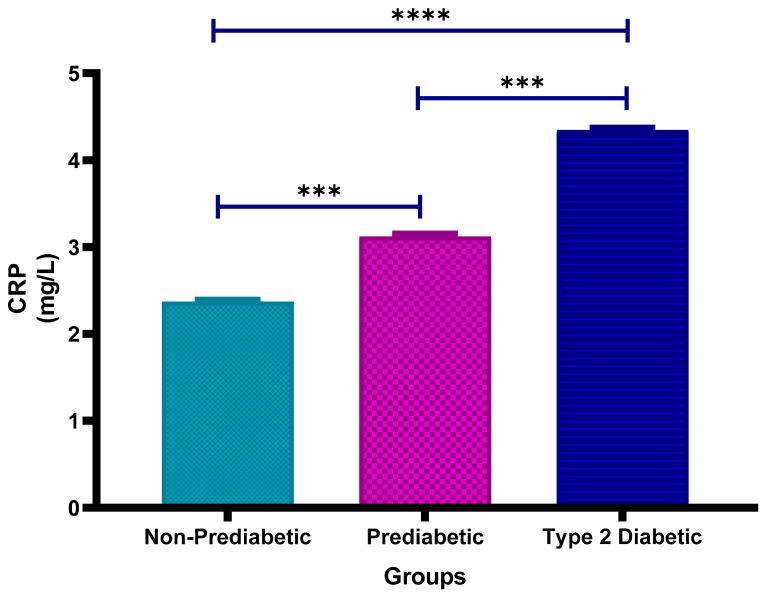
Plasma Hs-C-Reactive Protein (CRP) concentration in the non-prediabetic (NPD) group (*n* = 20), prediabetic (PD) group (*n* = 20) and type 2 diabetic (T2D) group (*n* = 20). Values are presented as means ± SEM. Asterisk Indicates the significant (*** *p* < 0.001, **** *p* < 0.0001) differences between the groups.

Figure 1 shows the plasma high-sensitivity C-Reactive Protein (CRP) concentrations in non-prediabetic (NPD), prediabetic (PD) and type 2 diabetic (T2D) individuals in Durban, South Africa. The mean ± SEM CRP concentrations were 2.37 ± 0.019 mg/L in the NPD group, 3.12 ± 0.031 mg/L in the PD group and 4.35 ± 0.025 mg/L in the T2D group. Statistical comparison tests revealed that CRP levels in the PD group were significantly higher than those in the NPD group (*p* = 0.0008). Furthermore, CRP concentrations in the T2D group were significantly higher than both the PD (*p* = 0.0008) and NPD (*p* < 0.0001) groups (See Figure 1). These findings indicate a progressive increase in CRP across the glycaemic spectrum. For context, Hs-CRP concentrations < 3 mg/L are generally considered to be within the normal range; thus, individuals in both the PD and T2D groups exhibited elevated CRP levels relative to clinical reference values [35].

### 3.3. Interleukin-6 (IL-6) Concentration

**Figure 2:**
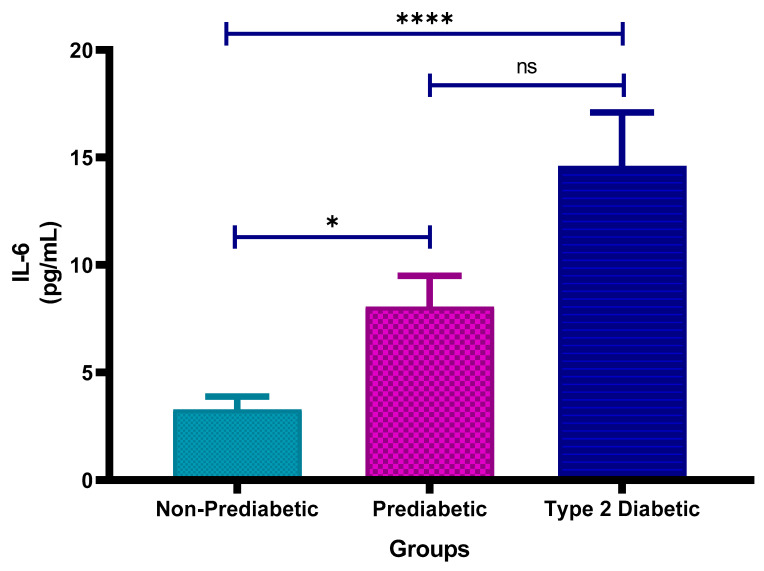
Interleukin-6 (IL-6) concentration in the non-prediabetic (NPD) group (*n* = 10), prediabetic (PD) group (*n* = 15) and type 2 diabetic (T2D) group (*n* = 15). Values are presented as means ± SEM. Asterisk Indicates the significant (* *p* < 0.05, **** *p* < 0.0001) differences between the groups. ns: Non-Significant.

Figure 2 shows the plasma Interleukin-6 (IL-6) concentrations in non-prediabetic (NPD), prediabetic (PD), and type 2 diabetic (T2D) individuals in Durban, South Africa. The mean ± SEM IL-6 concentrations were 3.28 ± 0.59 pg/mL in the NPD group, 8.06 ± 1.44 pg/mL in the PD group and 14.61 ± 2.49 pg/mL in the T2D group. Statistical comparisons test revealed that IL-6 levels in the PD group were significantly higher than those in the NPD group (*p* = 0.0369), while concentrations in the T2D group were significantly elevated compared to the NPD group (*p* < 0.0001). However, the difference between the PD and T2D groups was not statistically significant (*p* = 0.1648) (see Figure 2). These results indicate a trend of increasing IL-6 concentrations across the glycaemic spectrum. For reference, normal IL-6 concentrations in healthy individuals typically range between <1 to 5 pg/mL, suggesting that both the PD and T2D groups exhibited elevated IL-6 levels consistent with systemic inflammation [36].

### 3.4. Fibrinogen Concentration

**Figure 3:**
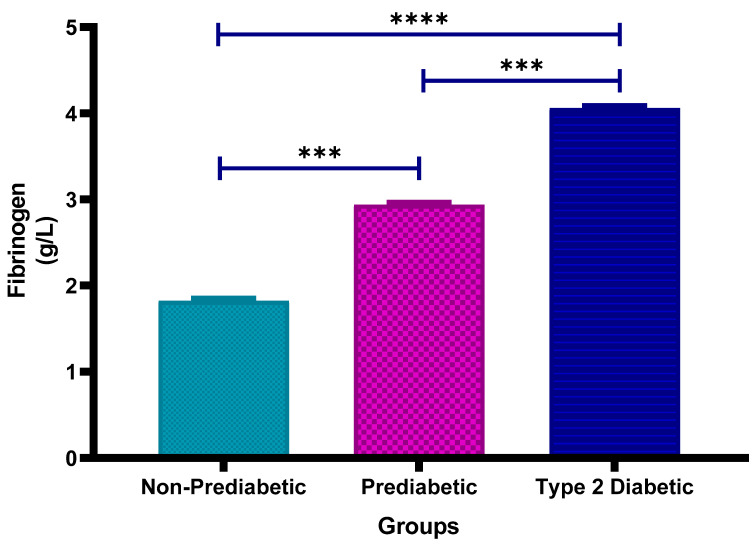
Plasma fibrinogen concentration in the non-prediabetic (NPD) group (*n* = 20), prediabetic (PD) group (*n* = 20) and type 2 diabetic (T2D) group (*n* = 20). Values are presented as means ± SEM. Asterisk Indicates the significant (*** *p* < 0.001, **** *p* < 0.0001) differences between the groups.

Figure 3 shows the plasma fibrinogen concentrations in non-prediabetic (NPD), prediabetic (PD) and type 2 diabetic (T2D) individuals in Durban, South Africa. The mean ± SEM fibrinogen concentrations were 1.823 ± 0.023 g/L in the NPD group, 2.938 ± 0.018 g/L in the PD group, and 4.060 ± 0.019 g/L in the T2D group. Statistical comparisons test showed that fibrinogen levels in the PD group were significantly higher than those in the NPD group (*p* = 0.0008) and significantly lower than those in the T2D group (*p* = 0.0008). Additionally, the T2D group had significantly higher fibrinogen concentrations compared to the NPD group (*p* < 0.0001) (see Figure 3). These results indicate a clear increase in fibrinogen levels across the glycaemic spectrum. For comparison, the typical plasma fibrinogen reference range in healthy adults is approximately 2–4 g/L, indicating that all study groups exhibited markedly elevated fibrinogen levels consistent with a pro-thrombotic or inflammatory state [35].

### 3.5. D-Dimer (D2D) Concentration

**Figure 4:**
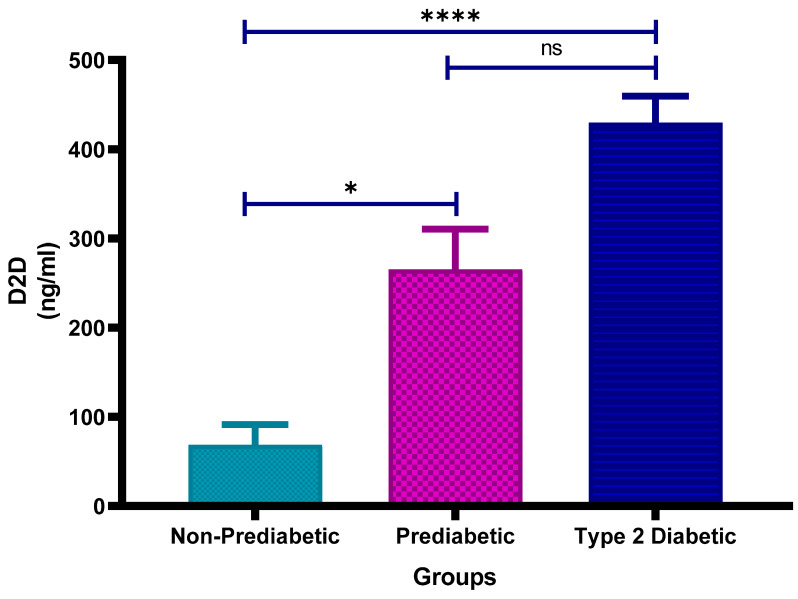
Plasma D-dimer (D2D) concentration in the non-prediabetic (NPD) group (*n* = 10), prediabetic (PD) group (*n* = 15) and type 2 diabetic (T2D) group (*n* = 15). Values are presented as means ± SEM. Asterisk Indicates the significant (* *p* < 0.05, **** *p* < 0.0001) differences between the groups. ns: Non-Significant.

Figure 4 shows the plasma D-dimer concentrations in non-prediabetic (NPD), prediabetic (PD), and type 2 diabetic (T2D) individuals in Durban, South Africa. The mean ± SEM D-dimer concentrations were 68.40 ± 22.88 ng/mL in the NPD group, 265.1 ± 45.11 ng/mL in the PD group, and 429.8 ± 29.82 ng/mL in the T2D group. Statistical comparisons test indicated that D-dimer levels in the PD group were significantly higher than those in the NPD group (*p* = 0.0451), while levels in the T2D group were significantly elevated compared to the NPD group (*p* < 0.0001). However, the difference between the PD and T2D groups was not statistically significant (*p* = 0.1009) (See Figure 4). These findings suggest a progressive increase in fibrinolytic activity across the glycaemic spectrum. For reference, normal plasma D-dimer concentrations are typically below 500 ng/mL [37]. Although the D-dimer levels in all groups in this study remained below this clinical threshold, individuals with T2D exhibited the highest concentrations, suggesting a trend toward increased coagulation and fibrinolytic activity.

### 3.6. Calcium Binding Protein (S100B) Concentration

**Figure 5:**
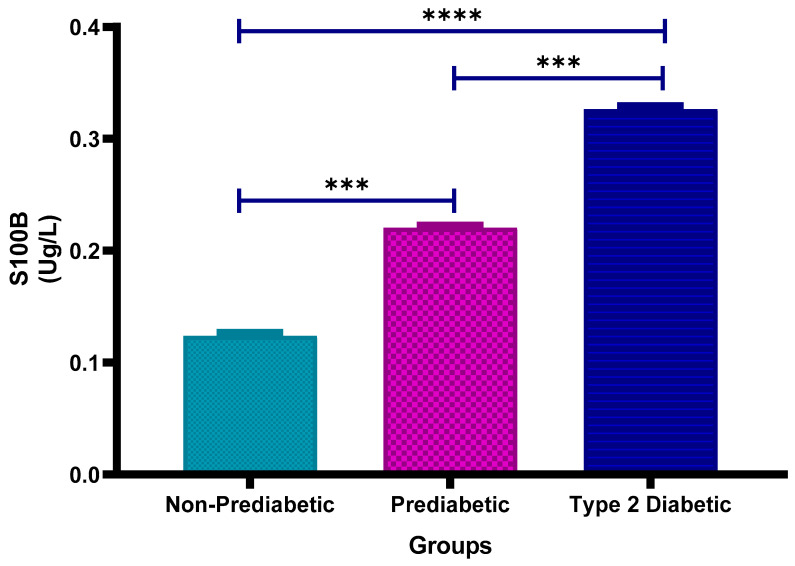
Plasma calcium binding protein (S100B) concentration in the non-prediabetic (NPD) group (*n* = 20), prediabetic (PD) group (*n* = 20) and type 2 diabetic (T2D) group (*n* = 20). Values are presented as means ± SEM. Asterisk Indicates the significant (*** *p* < 0.001, **** *p* < 0.0001) differences between the groups.

Figure 5 shows the plasma calcium-binding protein S100B concentrations in non-prediabetic (NPD), prediabetic (PD) and type 2 diabetic (T2D) individuals in Durban, South Africa. The mean ± SEM S100B levels were 0.124 ± 0.0031 Ug/L in the NPD group, 0.221 ± 0.0025 Ug/L in the PD group, and 0.327 ± 0.0033 Ug/L in the T2D group. Statistical comparisons test revealed that S100B concentrations were significantly higher in the PD group compared to the NPD group (*p* = 0.0008) and significantly lower in the PD group compared to the T2D group (*p* = 0.0008). Furthermore, the T2D group showed significantly higher S100B levels than the NPD group (*p* < 0.0001) (See Figure 5). These findings suggest a progressive increase in plasma S100B concentration with worsening glycaemic status. For reference, typical plasma S100B levels in healthy adults are below 0.15 Ug/L, indicating that individuals with PD or T2D exhibited elevated S100B consistent with possible neurovascular injury or inflammation [38].

### 3.7. Correlation Analysis

Analyses were conducted using Pearson’s correlation coefficients on the pooled dataset of all participants with available data (*n* = 60), combining the NPD, PD and T2D groups. Both impaired fasting glucose and HbA1c showed strong, positive correlations with S100B concentrations, with correlation coefficients of r = 0.75 and r = 0.75, respectively (*p* < 0.0001 for both; 95% CI: 0.62–0.84), as shown in Table 3. These significant positive correlations indicate that as blood glucose levels and long-term glycaemic control worsen, plasma S100B concentrations tend to increase. This suggests a link between glycaemic dysregulation and elevated S100B, which may reflect increased neurovascular injury or inflammatory processes in individuals progressing from non-prediabetic to diabetic states.

### 3.8. Glial Fibrillary Acidic Protein (GFAP) Concentration

**Figure 6:**
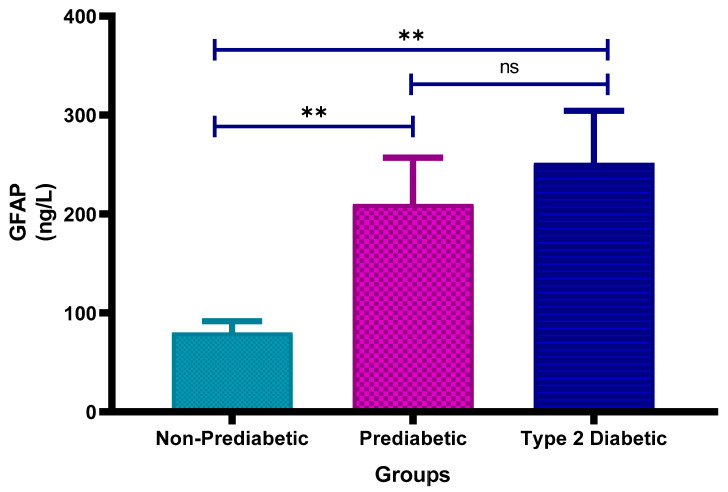
Plasma glial fibrillary acidic protein (GFAP) concentration in the non-prediabetic (NPD) group (*n* = 10), prediabetic (PD) group (*n* = 15) and type 2 diabetic (T2D) group (*n* = 15). Values are presented as means ± SEM. Asterisk Indicates the significant (** *p* < 0.01) differences between the groups. ns: Non-Significant.

Figure 6 shows the plasma glial fibrillary acidic protein (GFAP) concentrations in non-prediabetic (NPD), prediabetic (PD) and type 2 diabetic (T2D) individuals in Durban, South Africa. The mean ± SEM GFAP concentrations were 80.3 ± 11.3 ng/L in the NPD group, 210.1 ± 46.9 ng/L in the PD group and 251.7 ± 52.7 ng/L in the T2D group. Statistical comparisons test revealed that GFAP levels were significantly higher in the PD group compared to the NPD group (*p* = 0.0082) and significantly elevated in the T2D group compared to the NPD group (*p* = 0.0071). However, there was no significant difference between the PD and T2D groups (*p* > 0.9999) (See Figure 6). The reference range for serum GFAP varies by age and assay, but generally healthy adults (20–49) have levels ranging from 25 to 242 ng/L [39]. These findings suggest a rising trend in GFAP concentration with worsening glycaemic status. Notably, mean GFAP levels in the PD and T2D groups exceed the general adult reference range, providing supporting evidence of astrocyte activation and possible neurovascular impairment associated with dysglycaemia.

### 3.9. Neuron Specific Enolase (NSE) Concentration

**Figure 7:**
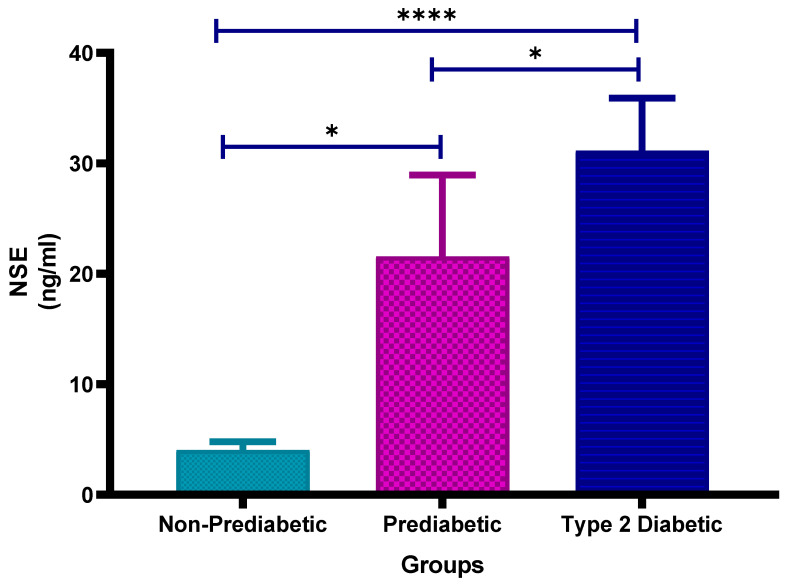
Neuron-specific enolase (NSE) concentration in the non-prediabetic (NPD) group (*n* = 10), prediabetic (PD) group (*n* = 15) and type 2 diabetic (T2D) group (*n* = 15). Values are presented as means ± SEM. Asterisk Indicates the significant (* *p* < 0.05, **** *p* < 0.0001) differences between the groups.

Figure 7 shows the plasma neuron-specific enolase (NSE) concentrations in non-prediabetic (NPD), prediabetic (PD) and type 2 diabetic (T2D) individuals in Durban, South Africa. The mean ± SEM NSE concentrations were 4.06 ± 0.73 ng/mL in the NPD group, 21.57 ± 7.39 ng/mL in the PD group and 31.16 ± 4.74 ng/mL in the T2D group. Statistical comparisons test revealed that NSE levels were significantly higher in the PD group compared to the NPD group (*p* = 0.0118), significantly elevated in the T2D group compared to the NPD group (*p* < 0.0001) and also significantly increased in the T2D group compared to the PD group (*p* = 0.0105) (see Figure 7). The reference range for serum NSE is typically 0–18.9 ng/mL [40]. The values observed in the PD and T2D groups exceed this range, suggesting progressive neuronal injury or neurovascular stress associated with dysglycaemia.

## 4. Discussion

The increasing prevalence of T2DM has been strongly linked to modern lifestyle factors, including poor dietary habits, physical inactivity and rapid urbanisation associated with economic development [41]. Research has demonstrated that many of the complications related to T2DM, such as cerebrovascular diseases, specifically stroke, begin during the prediabetic stage [42]. Despite this, there is limited understanding of the specific link between prediabetes and stroke risk, especially in populations where T2DM is on the rise. This study aimed to fill this gap by investigating the relationship between prediabetes and stroke risk in individuals aged 25–45 years from Durban, South Africa. Through the retrospective analysis of biomarkers related to inflammation, coagulation and brain damage, we sought to identify early indicators that could help predict stroke risk in prediabetic individuals and contribute to better detection and intervention strategies. It is important to note that this study did not directly assess clinical stroke events. Rather, biomarkers associated with inflammation, coagulation and neurovascular injury were used as surrogate indicators to explore potential stroke risk. This distinction is critical to prevent overinterpretation of the findings.

The results presented here are descriptive and based on biomarker trends rather than confirmed clinical stroke events. Therefore, the findings should be interpreted as indicative of potential stroke risk rather than deterministic outcomes. This study provides proof-of-concept evidence of early neurovascular, inflammatory and coagulation changes in individuals with prediabetes in a South African population—a group underrepresented in current research. Despite the limited sample size, the consistent trends across multiple biomarkers support the biological relevance of these findings. These results provide initial evidence that can inform and guide larger-scale future studies.

It should be acknowledged that the number of participants in each group was small, and the sex distribution was unequal. The primary objective of this study was to assess biomarker changes associated with prediabetes, rather than to evaluate sex-specific differences. Pooling data allowed for greater statistical power in addressing this research aim. However, participants were generally healthy young adults without major comorbidities, which supports the validity of pooling results across sexes in this study.

These results of this study support the growing understanding that prediabetes is not a benign intermediate state, but a metabolically active condition marked by early pathological changes. By highlighting significant biomarker alterations in a young South African cohort, this study contributes valuable regional data to a field largely shaped by research from other countries.

Insulin resistance is a key feature of prediabetes and plays a significant role in the development of associated complications, including stroke [29]. In individuals with prediabetes, insulin resistance results from defects in the insulin signalling pathways. These defects impair glucose uptake and metabolism, which can increase the risk of cardiovascular and cerebrovascular events [43]. One of the primary markers of glucose regulation in prediabetes is HbA1c, which reflects the average blood glucose levels over 3 months [44]. In this study, individuals in the PD group showed significantly elevated (*p* < 0.0001) HbA1c levels compared to the NPD group but significantly lower levels than that of individuals in the T2D group (*p* < 0.0001). This increase in HbA1c suggests ongoing hyperglycaemia and impaired glucose control, which is consistent with the definition of prediabetes as outlined by the ADA and WHO, where the condition is characterised by intermediate insulin resistance and elevated HbA1c levels [45]. The results in this study indicate that insulin resistance, as indicated by elevated HbA1c levels, may contribute to the increased risk of stroke in individuals with prediabetes. 

Inflammation is a fundamental physiological response that helps the body fight infections but when chronic, it can contribute to the development of various diseases, including cerebrovascular disease [46]. In a normal response, inflammation is acute, involving the activation of both immune and non-immune cells [46]. However, in prediabetes and T2DM, this inflammatory response becomes persistent and low-grade.

Chronic hyperglycaemia associated with prediabetes and T2DM stimulates the release of inflammatory cytokines such as CRP and IL-6. High levels of inflammatory cytokines have been known to cause injury to the vascular endothelium.

CRP is an acute-phase protein synthesised by the liver following inflammation, and it usually indicates systemic inflammation [47]. The high level of CRP was linked to an increased risk for rupture of plaque and vascular thrombosis since CRP mediates endothelial dysfunction and destabilises atherosclerotic plaques toward rupture [48]. Increased IL-6 levels are strongly associated with the development of chronic inflammation [49]. The pro-inflammatory cytokine IL-6 plays a role in several biological activities, including cell proliferation and differentiation and has been implicated in the pathogenesis of atherosclerosis and plaque formation within the vasculature [50]. In individuals with prediabetes, elevated blood glucose levels exacerbate this inflammatory response, leading to endothelial injury and the initiation of atherosclerosis, a major cause of ischaemic stroke [51]. This study’s results revealed significantly higher CRP levels in the PD group compared to NPD (*p* = 0.0008), and significantly higher levels in T2D compared to both PD (*p* = 0.0008) and NPD (*p* < 0.0001) groups. These results indicate a progressive increase in CRP across the glycaemic spectrum. For context, Hs-CRP concentrations below 3 mg/L are generally considered normal, so individuals in both PD and T2D groups showed elevated CRP relative to clinical reference values.

These CRP findings align with the conclusions drawn from previous research assessing various markers of carotid atherosclerosis in individuals with T2D [52]. The study demonstrated a significant correlation between elevated CRP levels and the presence of carotid artery disease, as well as an increased risk of cerebrovascular events in individuals with T2DM. However, by focusing on a younger South African prediabetic population, this study extends current knowledge to an underrepresented group, providing new insights into early inflammatory processes linked to stroke risk. The reviewed literature further emphasises CRP’s potential as a predictive marker for stroke risk. Additionally, it highlights T2DM as an independent risk factor for both stroke and its recurrence. The high levels of CRP in the present study strongly point toward its role as an inflammatory marker related to carotid atherosclerosis, one of the major contributors to ischaemic stroke risk. The higher level of CRP among the individuals with T2D further established the role of inflammation in the pathogenesis of carotid atherosclerosis as one of the key contributors to ischaemic stroke. The increased levels of CRP in prediabetes suggest that even minor early metabolic disturbances may enhance vascular risk, hence the stratification and need for early intervention in such cases.

Statistical analysis of this study’s results showed that IL-6 levels were significantly higher in the PD group compared to the NPD group (*p* = 0.0369) and significantly elevated in the T2D group compared to the NPD group (*p* < 0.0001). However, there was no significant difference between the PD and T2D groups (*p* = 0.1648). The IL-6 results further support the findings of [53,54], who found higher plasma IL-6 levels in individuals with diabetes compared to non-diabetic individuals. However, unlike the study by Pickup, which indicated no significant difference in IL-6 levels among individuals with diabetes and complications of macrovascular and microvascular nature, the study by [54], along with this current study, noted significant differences. Though this study’s IL-6 results align with the findings by [54], it has to be taken into consideration that another study has linked IL-6 with increased insulin resistance, further exacerbating hyperglycaemia and vascular status [55]. This creates a vicious cycle, increasing the risk of stroke in individuals with T2DM. Overall, CRP and IL-6 levels and consequently the risk of a stroke incrementally increases from the NPD to PD to T2D group, as a result of worsening metabolic and inflammatory profiles, with prediabetes representing an intermediate stage of the inflammatory response. The CRP and IL-6 results follow a similar trend as seen in the HbA1C results, which increased incrementally from the NPD to PD to T2D group.

Although individuals with HIV were excluded from this study to reduce confounding, it is important to acknowledge that HIV is highly prevalent in KwaZulu-Natal and can independently elevate inflammatory markers such as CRP and IL-6. As such, while the exclusion enhances the specificity of our findings, it may limit the generalisability of results to populations with high HIV comorbidity. HIV and other major illnesses are not the only potential confounding factors; lifestyle-related variables such as smoking, alcohol use and poor diet may also influence inflammatory and vascular biomarkers. These factors were not accounted for in this study.

It is important to note that unassessed lifestyle factors, including smoking, alcohol consumption, diet, and body mass index (BMI), could influence inflammatory and coagulation biomarkers. Smoking and excessive alcohol intake are known to elevate CRP and fibrinogen levels, potentially confounding the observed biomarker elevations in prediabetic and diabetic individuals [56,57]. Similarly, higher BMI and poor dietary habits can exacerbate low-grade inflammation and hypercoagulability, contributing to increased vascular risk [58,59]. In future research, multivariate adjustments could be applied by including lifestyle factors as covariates in regression models (e.g., adjusting CRP levels for smoking status or BMI), which would help distinguish the independent contribution of prediabetes from these confounders. Therefore, future studies should consider incorporating these variables and performing multivariate adjustments to more accurately assess the independent effects of prediabetes on stroke-related biomarkers, particularly in populations with high HIV prevalence or diverse lifestyle exposures.

Beyond its role in driving inflammation, T2DM is also known to impair the normal functioning of the coagulation cascade, further enhancing the risk of thrombotic events. The inflammatory markers not only indicate systemic inflammation but also contribute to the activation of coagulation pathways, leading to an increased thrombotic state [60].

Fibrinogen and D-dimer are key biomarkers involved in the coagulation cascade and thrombus formation [61]. Fibrinogen is a plasma protein that plays a critical role in blood clot formation, while D-dimer is a degradation product of fibrin, typically elevated in cases of thrombosis [62]. Both are associated with increased thrombogenic activity, contributing to the risk of cardiovascular and cerebrovascular events [63,64]. Elevated fibrinogen is also associated with pro-inflammatory states, which further promotes vascular dysfunction [65]. In T2DM, insulin resistance predisposes individuals to abnormal fibrinogen metabolism, promoting clot formation and impairing fibrinolysis [66]. In addition, raised D-dimer levels in individuals with diabetes indicate ongoing thrombotic activity and enhanced vascular events [67]. The results in this study indicated that plasma fibrinogen levels were significantly higher in PD than in NPD (*p* = 0.0008) and further elevated in T2D compared to both PD (*p* = 0.0008) and NPD (*p* < 0.0001). These findings demonstrate a stepwise increase in fibrinogen along the glycaemic spectrum.

While fibrinogen levels remained within clinically accepted reference ranges (2–4 g/L), the observed upward trend across the glycaemic spectrum may represent an early, compensatory response to low-grade inflammation or endothelial activation. In the absence of acute illness or overt vascular damage, fibrinogen concentrations may rise gradually but remain subclinical, supporting its role as a sensitive marker of early prothrombotic risk in dysglycaemic individuals. Fibrinogen is an acute-phase reactant, and its levels can remain in the “normal” range until systemic inflammation or endothelial injury reaches a more advanced or clinically overt stage. In early dysglycaemia, like prediabetes, subtle inflammatory or metabolic disturbances may stimulate fibrinogen production modestly, but not enough to push levels beyond clinical thresholds.

The results in the study indicated that D-dimer levels were significantly higher in PD vs. NPD (*p* = 0.0451) and in T2D vs. NPD (*p* < 0.0001), though the PD–T2D difference was not significant (*p* = 0.1009). Similarly to fibrinogen, D-dimer levels remained within the reference range but showed a stepwise increase across the glycaemic spectrum. This concurrent elevation suggests active clot formation and breakdown, which is characteristic of a hypercoagulable state. Fibrinogen reflects the production of fibrin and the potential for clot formation, while D-dimer indicates the breakdown of fibrin clots. The simultaneous rise in both markers suggests that coagulation is being activated alongside ongoing fibrinolysis, likely driven by persistent endothelial dysfunction, vascular inflammation, and chronic metabolic stress in individuals with dysglycaemia.

The fibrinogen and D-dimer levels observed in this study were significantly elevated across the glycaemic spectrum but remained within clinically accepted reference ranges. This finding contrasts with several international studies reporting elevations beyond these limits in diabetes mellitus. By demonstrating early, subclinical increases in these coagulation markers in a younger South African prediabetic population, the study provides novel evidence of a prothrombotic state that may precede overt vascular disease, thereby enhancing understanding of coagulation dynamics in early dysglycaemia.

While inflammation and coagulation are common pathways in both diabetes and stroke pathology, these are non-specific mechanisms. As chronic inflammation and coagulation abnormalities persist in prediabetes and T2DM, they impair vascular function, heightening the risk of cerebrovascular damage. These changes contribute to the development of brain injury, particularly ischaemic stroke, by compromising cerebral circulation. Therefore, assessing brain injury provides the most accurate insight into the impact of these metabolic disturbances in this study.

S100B is a protein with diverse functions in the body’s normal physiology [68]. This protein is predominantly found in the central nervous system and is released when there is damage to neural or glial cells, reflecting brain injury or neurological distress [69]. S100B is mainly produced by astrocytes and is involved in regulating processes like cell proliferation, differentiation and inflammation [70].

Hyperglycaemia accelerates intracellular glucose metabolism and mitochondrial electron transport, promotes the formation of advanced glycation end products, and activates NADPH oxidase, all of which increase the generation of reactive oxygen species (ROS) [68]. Elevated ROSs induce oxidative stress within the neurovascular unit and act as potent activators of astrocytes, triggering morphological and functional changes via intracellular signalling pathways [70]. In response to this oxidative and inflammatory environment, activated astrocytes upregulate and release calcium-binding proteins such as S100B into the extracellular space [69]. Once extracellular, S100B functions both as a damage-associated molecular pattern and as a mediator of neuroinflammation, linking hyperglycaemia-driven oxidative stress to astrocytic activation and S100B secretion [68,69,70].

In individuals with T2DM, S100B levels are often elevated with several contributing factors [24]. These include chronic low-grade inflammation, oxidative stress and microvascular changes, which can result from prolonged hyperglycaemia and metabolic abnormalities. Elevated S100B levels can indicate neurological complications associated with the disease, such as cognitive impairment, diabetic neuropathy or even an increased risk of stroke [24]. Statistical comparisons of results in this study revealed significantly higher S100B concentrations in the PD group compared to the NPD group (*p* = 0.0008), and significantly higher levels in the T2D group compared to both the NPD group (*p* < 0.0001) and the PD group (*p* = 0.0008). These findings demonstrate a stepwise elevation in plasma S100B concentrations with worsening glycaemic status. For reference, typical plasma S100B levels in healthy adults are below 0.15 µg/L. This significant difference can be attributed to factors such as persistent hyperglycaemia and associated conditions within the T2D group, indicating that individuals with type 2 diabetes suffered the greatest extent of brain injury, with the NPD levels signifying a healthier and less injured state. The elevated level of S100B in the PD group can be attributed to the fact that individuals with prediabetes often experience intermediate insulin resistance, chronic inflammation and metabolic dysfunction. These factors can lead to small vessel damage in the brain, which may eventually result in strokes. Elevated S100B levels may not directly predict an imminent stroke event; however, it may serve as a marker of neurological damage that has occurred, reflecting the severity of the event [71]. S100B is more indicative of a past or ongoing stroke rather than a predictive marker for future stroke risk. The incremental rise in S100B levels from the NPD to PD to T2D group reflects the increasing severity of brain injury, with prediabetes serving as an intermediary stage. This trend is consistent with the incremental increase pattern observed in the HbA1c, CRP, IL-6, fibrinogen and D-dimer levels. Moreover, the correlation analysis revealed a strong positive correlation between both impaired fasting blood glucose and HbA1c with the calcium-binding protein S100B in the NPD, PD and T2D groups. The small *p*-values (0.0001) indicate that these correlations are statistically significant, implying that as glucose and HbA1c levels increase, S100B levels tend to rise.

GFAP and NSE are important biomarkers used to assess brain injury, especially in neurological disorders like stroke [72,73,74]. GFAP is primarily found in the astrocytes and its increased level reflects the activation or injury of astrocytes that may result from inflammation or damage to the brain [75]. As a neuroinflammatory marker, GFAP has also been used to assess the extent of brain damage in cases of stroke or neurodegenerative conditions [76,77]. NSE, in turn, is an intracellular enzyme highly expressed in neurons, where elevated levels are indicative of neuronal damage or death [78]. Both biomarkers are valuable in assessing neuronal injury and have been involved in the process of determining the effects of chronic disorders, such as T2DM, on the brain. In individuals with prediabetes or T2DM, elevated levels of GFAP and NSE can indicate early neuronal damage, providing insight into the risk of cerebrovascular events, including stroke.

The results in this study indicated that the mean ± SEM NSE concentrations were 4.06 ± 0.73 ng/mL in the NPD group, 21.57 ± 7.39 ng/mL in the PD group, and 31.16 ± 4.74 ng/mL in the T2D group. Statistical comparisons revealed that NSE concentrations were significantly higher in the PD group compared to the NPD group (*p* = 0.0118), significantly elevated in the T2D group compared to the NPD group (*p* < 0.0001) and significantly increased in the T2D group compared to the PD group (*p* = 0.0105). These results indicate a stepwise rise in NSE concentrations with worsening glycaemic status. Values observed in the PD and T2D groups exceeded the reference range, suggesting the presence of progressive neuronal injury or neurovascular stress linked to dysglycaemia.

The NSE findings of this study are consistent with the work by [23], who reported concentrations of 7.5 ± 1.5 ng/mL in controls, 15.2 ± 2.4 ng/mL in normoglycaemic stroke patients, and 19.7 ± 4.7 ng/mL in hyperglycaemic stroke patients. Quantitatively, NSE in our PD group (21.57 ± 7.39 ng/mL) was approximately 1.4-fold higher than in hyperglycaemic stroke patients, while concentrations in the T2D group (31.16 ± 4.74 ng/mL) were nearly 1.6-fold greater. Even at the prediabetic stage, NSE levels were almost three times higher than the non-stroke control group reported by [23] (21.57 vs. 7.5 ng/mL). These findings demonstrate a consistent pattern of increased neuronal injury associated with glycaemic dysregulation and suggest that neuronal stress of a magnitude similar to, or greater than, that observed in stroke may already be present in prediabetes. This supports the hypothesis that hyperglycaemia is not only a consequence of acute cerebrovascular events but may act as an early driver of neurovascular injury, thereby predisposing individuals to future stroke.

These results further support NSE as a reliable biomarker of neuronal injury, indicating that hyperglycaemia in prediabetic and diabetic individuals can induce metabolic stress and early neurovascular damage, even in the absence of clinical stroke.

The results in this study revealed that GFAP levels were significantly higher in the PD group compared to the NPD group (*p* = 0.0082) and significantly elevated in the T2D group compared to the NPD group (*p* = 0.0071). However, there was no significant difference between the PD and T2D groups (*p* > 0.9999). The reference range for serum GFAP varies by age and assay, but generally, healthy adults aged 20 to 49 years have levels ranging from 25 to 242 ng/L. The GFAP findings of this study are consistent with the work by [22], who examined serum levels of GFAP in individuals with T2DM and cognitive impairments. The study found that individuals with T2DM, particularly those with cognitive impairments, had significantly higher GFAP levels compared to healthy controls. While [22] focused on GFAP and cognitive impairments, GFAP is also considered an important marker in stroke assessment. This supports the notion that elevated GFAP reflects astrocyte activation and neurovascular injury that may underlie both cognitive decline and increased stroke risk in dysglycaemic states. In this study, which investigated stroke risk specifically in individuals with prediabetes and no other underlying disorders or diseases, similar elevated GFAP levels were observed. This supports the relevance of GFAP as a biomarker in assessing brain injury and differentiating between stroke types, highlighting its significance in individuals with diabetes at risk for cerebrovascular events.

The neurovascular injury markers S100B, GFAP and NSE, which were the central focus of this study, showed significant elevations across the glycaemic spectrum, providing strong evidence of early neurovascular compromise. These findings suggest that subclinical blood–brain barrier disruption and neuronal stress may already be present in the prediabetic state. Taken together with the elevations in inflammation and coagulation, these results suggest that even in the absence of clinical stroke, individuals with prediabetes may exhibit early signs of neuroinflammation and neuronal injury. Supporting evidence from previous research shows that white matter alterations are significantly correlated with cognitive impairment, particularly in processing speed, in individuals with Metabolic Syndrome [79]. These findings indicate that a cluster of silent vascular risk factors may contribute to progressive cerebrovascular pathology and cognitive decline as patients age. This underscores the relevance of our study, as identifying early biomarker changes in the prediabetic stage could help detect and mitigate subclinical neurovascular alterations before irreversible structural or cognitive damage occurs. This provides a biological rationale for incorporating neurological screening in metabolic disease monitoring, particularly in high-risk populations. If left untreated, this covert neurovascular injury could potentiate cumulative damage, ultimately elevating the risk of symptomatic cerebrovascular events such as stroke as metabolic dysfunction worsens. Furthermore, given South Africa’s rising burden of non-communicable diseases and the relatively young age of onset observed in this cohort (25 to 45 years), these findings highlight the urgent need for early detection strategies that integrate neurological risk profiling into routine metabolic health assessments.

The neurovascular injury markers, taken together with inflammatory and coagulation markers, suggest that biological processes linked to stroke pathogenesis may begin much earlier than traditionally expected. The findings may inform future research on predictive biomarker panels for stroke risk stratification in younger adults, particularly in regions like South Africa, where the burden of diabetes-related complications is rising.

## 5. Conclusions

Intermediate insulin resistance and intermediate hyperglycaemia associated with prediabetes lead to inflammation and endothelial dysfunction, which are known risk factors for stroke. There is a positive correlation between insulin resistance and inflammatory markers, such as CRP and IL-6, suggesting that prediabetes increases the risk of developing cerebrovascular events. While there are several other markers of brain injury, this study demonstrated that S100B, GFAP and NSE have potential for use as biomarkers in the development of prediabetes-associated stroke events. The elevated neurodegenerative markers (S100B, GFAP, and NSE) in both the prediabetes (PD) and T2D groups indicate ongoing neurovascular damage and an increased stroke risk that begins to emerge in the prediabetic stage and intensifies with progression to T2DM. These results suggest that individuals with prediabetes and T2DM experience early stages of neurodegeneration even in the absence of a stroke. Importantly, prediabetes is a reversible condition, and early identification of at-risk individuals provides a critical window for intervention through lifestyle modification and glycaemic control. The panel of biomarkers used, while requiring further independent validation, demonstrates potential as a cost-effective, non-invasive tool for early stroke risk monitoring by detecting intermediate metabolic and vascular alterations that precede overt cerebrovascular events. These findings provide novel, region-specific insights from a South African cohort, highlighting the burden of prediabetes in local populations and emphasising the potential for early detection, preventive strategies, and biomarker-based monitoring in communities underrepresented in current research. The current study, therefore, warrants further investigation on these markers for the early detection of stroke risk in prediabetes. However, it must be emphasised that this study did not include participants with confirmed stroke diagnoses, and the findings are based solely on biomarker trends suggestive of elevated risk; therefore, findings should be interpreted as indicative of potential stroke risk rather than deterministic outcomes. The study’s sample pool was limited to two hospitals in Durban, so findings may not be generalisable across the entire South African population. Due to the retrospective nature of the study, it also did not account for lifestyle factors like smoking, alcohol or diet. Although the sample size was limited, the findings remain clinically significant and highlight the potential utility of the biomarker panel for early stroke risk monitoring and preventive strategies. The results provide valuable initial evidence to inform and guide larger-scale, prospective investigations in the future. Future research should include these variables, expand the sample size and include diverse populations. Longitudinal studies are needed to clarify the cause-and-effect relationship between prediabetes and stroke risk. A key recommendation is to incorporate advanced diagnostic techniques, such as MRI and Transcranial Doppler Ultrasound, to improve the accuracy of stroke detection and validate biomarkers like S100B, GFAP and NSE as non-invasive stroke indicators. Additionally, studies should investigate individuals who have already experienced a stroke, monitoring their glucose and insulin levels to assess whether prediabetes or insulin resistance contributed to the stroke. This could provide valuable insights into the role of metabolic disturbances in stroke development and recurrence, ultimately informing targeted prevention and treatment strategies.

Furthermore, South Africa as a whole needs to raise awareness about diabetes, particularly prediabetes and its associated risks. While it may not receive the same attention as infectious diseases, such as HIV/AIDS or tuberculosis, diabetes complications, such as stroke, cardiovascular disease and kidney failure, can be just as fatal. Public health campaigns should educate the population about early detection, lifestyle changes and diabetes management to reduce its impact, ultimately improving health outcomes across the country.

## Figures and Tables

**Table 1:** American Diabetes Association Criteria.

Fasting Plasma Glucose (IFG)	2 Hour Plasma Glucose (IGT)	HbA1c%
5.6–6.9 mmol/L	7.8–11.0 mmol/L	5.7–6.4%

**Table 2:** Demographic and clinical characteristics of the study participants in the non-prediabetic, prediabetic and type 2 diabetic groups. Data are presented as mean (95% CI) or median (IQR), depending on the normality and proportion of variables.

	Non-Prediabetic	Prediabetic	Type 2 Diabetic
Age (mean)	36.13 (33.62–38.64)	38.91 (37.08–40.75)	40.34 (38.69–41.99)
Sex			
Male	12	20	11
Female	18	15	24
n	30	35	35
Impaired fasting glucose	5.95 mmol/L	7.1 mmol/L	11.6 mmol/L
median (IQR)	(5.7–6.2)	(6.8–7.5)	(9.4–14.9)
HbA1c% mean	5.35% (5.29–5.41)	6.18% (6.04–6.31)	9.72% (8.80–10.64)

**Table 3:** Correlation between glucose and glycated haemoglobin (HbA1c) with calcium-binding protein (S100B) in the non-prediabetic (NPD), prediabetic (PD) and type 2 diabetic (T2D) groups. r = Pearson’s correlation coefficient, *n* = sample size. 95% confidence intervals (CI) for each correlation are included.

Correlation Analysis	Impaired Fasting Glucose vs. S100B	HbA1c vs. S100B
r	0.7475	0.7458
95% CI	0.6093–0.8417	0.6068–0.8406
*p* value	0.0001	0.0001
*n*	60	60

## Data Availability

Upon request, the raw data supporting the conclusions of this article will be made available by the authors.

## Abbreviations

The following abbreviations are used in this manuscript:

ADA	American Diabetes Association
BREC	Biomedical Research Ethics Committee
CI	Confidence Level
CRP	C-reactive protein
CT	Computed tomography
ELISA	Enzyme-Linked Immunosorbent Assay
GFAP	Glial fibrillary acidic protein
HbA1c	Glycated Haemoglobin
IFG	Impaired fasting glucose
IGT	Impaired glucose tolerance
IL-6	Interleukin 6
IQR	Interquartile Range
MRI	Magnetic resonance imaging
NPD	Non Prediabetic
NSE	Neuron-specific enolase
PD	Prediabetic
ROS	Reactive oxygen species
S100B	Calcium-binding protein B
SEM	Standard error of mean
T2D	Type 2 diabetic
T2DM	Type 2 diabetes mellitus
UKZN	University of KwaZulu-Natal
WHO	World Health Organization
